# Structured clinical adjudication of *Streptomyces* spp. recovered from bronchoalveolar lavage in severe COVID-19 ARDS complicated by polymicrobial lower respiratory tract infection during invasive mechanical ventilation: a case report

**DOI:** 10.3389/fmed.2026.1880981

**Published:** 2026-08-27

**Authors:** Killen H. Briones-Claudett, Jaime Benites Solís, María Fernanda Pico Mendoza, Pedro Barberan-Torres, Diana C. Briones Márquez, Catherine Parreno, Ivonne Asqui, Anahí D. Briones-Zamora, Killen H. Briones-Zamora

**Affiliations:** 1 Universidad de Especialidades Espíritu Santo, Samborondón, Ecuador; 2 Briones PulmoCare, Medical Education and Research Group, Guayaquil, Ecuador; 3 Universidad de Guayaquil, Guayaquil, Ecuador; 4 Omni Hospital, Guayaquil, Ecuador; 5 Universidad Internacional del Ecuador (UIDE), Quito, Ecuador; 6 Hospital Clínica Panamericana, Guayaquil, Ecuador; 7 Interhospital S.A., Guayaquil, Ecuador; 8 Instituto Ecuatoriano de Seguridad Social (IESS), Guayaquil, Ecuador; 9 Instituto Universitario Italiano de Rosario (IUNIR), Rosario, Argentina

**Keywords:** acute respiratory distress syndrome, antimicrobial stewardship, bronchoalveolar lavage, COVID-19, lower respiratory tract infection, multiplex PCR, *Streptomyces* spp., clinical adjudication

## Abstract

**Background:**

Lower respiratory tract infections developing during invasive mechanical ventilation in patients with severe COVID-19 acute respiratory distress syndrome (ARDS) present important diagnostic and therapeutic challenges. *Streptomyces* spp., an uncommon respiratory isolate, frequently raises uncertainty regarding contamination, colonization, or clinically relevant infection because standardized criteria for clinical interpretation are lacking.

**Case presentation:**

A 65-year-old woman with hypertension and obesity was admitted with severe COVID-19 ARDS requiring invasive mechanical ventilation after failure of high-flow nasal cannula. Baseline laboratory evaluation demonstrated leukocytosis (15.65 × 10^3^/μL), neutrophilia (14.73 × 10^3^/μL), lymphopenia (0.92 × 10^3^/μL; neutrophil-to-lymphocyte ratio 16), C-reactive protein 134 mg/L, ferritin 2,130 ng/mL, and procalcitonin 0.5 ng/mL. Bronchoscopy performed approximately 24–36 h after intubation revealed purulent secretions, and quantitative bronchoalveolar lavage culture recovered methicillin-resistant *Staphylococcus aureus*, CTX-M–positive *Klebsiella pneumoniae*, and *Streptomyces* spp. The BioFire FilmArray^®^ Pneumonia Panel confirmed MRSA and *K. pneumoniae* with CTX-M detection but does not include *Streptomyces* spp. A structured clinical adjudication process integrating specimen quality, microbiological findings, quantitative culture, host characteristics, radiological assessment, therapeutic context, and clinical evolution supported interpretation of *Streptomyces* spp. as most compatible with colonization rather than invasive pulmonary infection. Antimicrobial therapy was optimized with meropenem and linezolid according to the confirmed bacterial pathogens, without additional organism-specific therapy directed against *Streptomyces*.

**Conclusion:**

This case illustrates the application of a structured clinical adjudication process to interpret recovery of an uncommon respiratory microorganism under conditions of microbiological uncertainty. Integration of quantitative bronchoalveolar lavage culture, rapid multiplex PCR, host factors, radiological assessment, and clinical evolution may support antimicrobial optimization while avoiding overinterpretation of uncommon respiratory isolates. The proposed approach should be regarded as an educational and descriptive clinical illustration requiring evaluation in future studies.

## Introduction

Severe COVID-19 ARDS is complicated by secondary bacterial infections in 20%–35% of ICU admissions. Lower respiratory tract infections during invasive mechanical ventilation are frequent in critically ill patients with COVID-19 ARDS and may involve polymicrobial microbiological findings that complicate therapeutic decision-making (1–3).

*Streptomyces* spp. are aerobic actinomycetes ubiquitous in soil and water (4). Although invasive *Streptomyces* infections have been described, particularly in immunocompromised hosts, the recovery of *Streptomyces* spp. from respiratory specimens frequently creates uncertainty regarding contamination, colonization, or clinically relevant infection (5, 6).

In clinical practice, interpretation of uncommon microorganisms recovered from respiratory specimens often requires integration of microbiological, radiological, therapeutic, and host-related information, particularly when polymicrobial infection and prior antimicrobial exposure complicate attribution of pathogenic significance (5). Quantitative bronchoalveolar lavage culture and rapid multiplex PCR may provide timely microbiological and resistance-marker information to support antimicrobial decision- making; however, these tools do not independently establish the pathogenic role of uncommon organisms detected in polymicrobial samples (7).

Here, we present a case of severe COVID-19 ARDS complicated by polymicrobial lower respiratory tract infection during invasive mechanical ventilation, in which *Streptomyces* spp. was recovered from bronchoalveolar lavage. The report illustrates a structured clinical adjudication process used to integrate microbiological findings, host characteristics, radiological assessment, antimicrobial therapy, and clinical progression to support targeted antimicrobial optimization under conditions of microbiological uncertainty.

## Case presentation

### Patient presentation and initial course

A 65-year-old woman presented to the emergency department with an 8-day history of fever (38.9 °C), headache, arthralgia, and progressive dyspnea. On admission, she was tachypneic (30 breaths/min) and tachycardic (115 beats/min). Her medical history included hypertension treated with amlodipine, obesity (body mass index 33 kg/m^2^), and a remote history of cervical adenocarcinoma treated 20 years earlier with chemoradiotherapy. Oncology follow-up 6 months prior showed no evidence of active malignancy.

**FIGURE 1:**
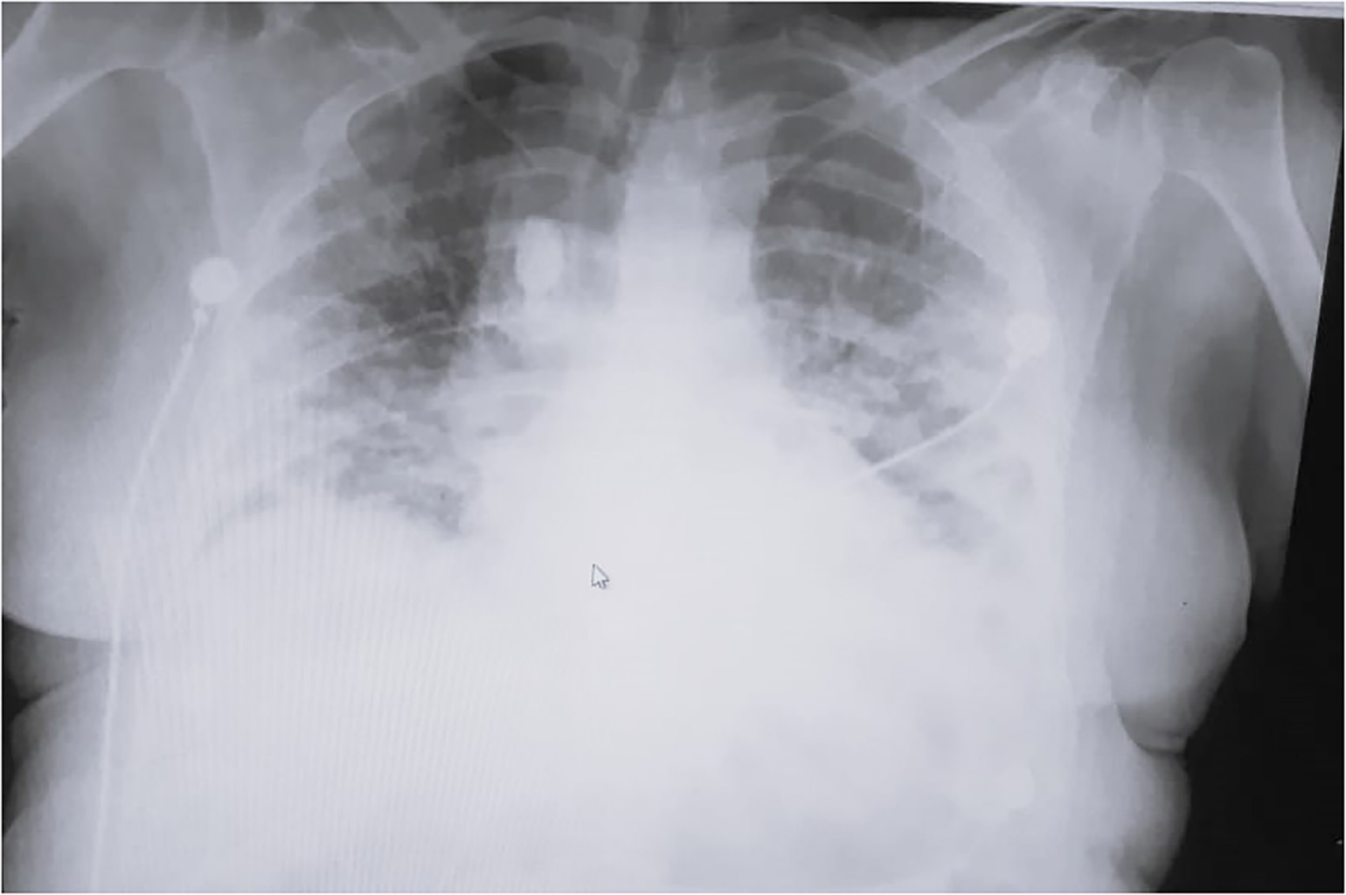
Portable anteroposterior chest radiograph on hospital admission. Portable anteroposterior (AP) chest radiograph obtained on hospital admission before initiation of invasive mechanical ventilation. The image demonstrates bilateral diffuse patchy air-space opacities predominantly involving the mid and lower lung fields, consistent with severe COVID-19 pneumonia. No pleural effusion or pneumothorax is evident on this radiograph. These imaging findings provided the initial radiological context for ICU admission and subsequent invasive mechanical ventilation.

SARS-CoV-2 infection was confirmed by RT-PCR. A portable anteroposterior chest radiograph obtained on hospital admission before initiation of invasive mechanical ventilation demonstrated bilateral diffuse patchy air-space opacities predominantly involving the mid and lower lung fields, consistent with severe COVID-19 pneumonia, without pleural effusion or pneumothorax (Figure 1). She was admitted to the ICU with ARDS secondary to COVID-19 pneumonia. High-flow nasal cannula was initiated (FiO_2_ 0.80). Initial therapy included dexamethasone (6 mg/day), remdesivir (200 mg loading dose followed by 100 mg/day), enoxaparin (40 mg subcutaneously once daily), and atorvastatin (40 mg/day). Empiric antimicrobials were started with piperacillin/tazobactam (4.5 g IV every 6 h) plus moxifloxacin (400 mg once daily) (ICU days 1–3). Key ICU events and management milestones are summarized in Table 1. Laboratory evaluation before ICU admission demonstrated leukocytosis (15.65 × 10^3^/μL) with marked neutrophilia (absolute neutrophil count 14.73 × 10^3^/μL) and lymphopenia (absolute lymphocyte count 0.92 × 10^3^/μL; neutrophil-to-lymphocyte ratio 16). Inflammatory biomarkers were markedly elevated, including C-reactive protein 134 mg/L, ferritin 2,130 ng/mL, and procalcitonin 0.5 ng/mL, consistent with severe systemic inflammation associated with COVID-19.

**TABLE 1:** ICU and hospital timeline: key clinical events.

Timeline	Clinical event	Vital signs/gas exchange	Key intervention/decision
ICU day 1	ICU admission for COVID-19 ARDS. HFNC initiated FiO2 0.80. Started dexamethasone 6 mg/day, remdesivir, enoxaparin, atorvastatin. Empiric PTZ + moxifloxacin initiated.	SpO_2_ ∼88% on HFNC FiO2 0.80; HR 110 bpm; RR 28	Supportive care; empiric broad-spectrum antibiotics
ICU day 2	HFNC failure (PaO_2_ 55 mmHg on FiO_2_ 1.0). Orotracheal intubation and invasive mechanical ventilation (SIMV mode). Sedation with propofol + fentanyl.	PaO_2_ 55 mmHg, FiO_2_ 1.0 (pre-intubation); Post-intubation SpO_2_ 92% on FiO_2_ 0.60, PEEP 10	Intubation for worsening hypoxemic respiratory failure
ICU day 3 a.m.	Flexible bronchoscopy performed. Findings: erythematous mucosa, purulent secretions bilateral, RUL mucus plug obstructing right main bronchus (removed). BAL obtained.	No immediate change; awaiting diagnostic results	Diagnostic bronchoscopy; BAL sent for quantitative culture + FilmArray
ICU day 3 p.m.	FilmArray (BAL) result returned: MRSA (mecA), ESBL-*Kp* (ESBL marker), NO carbapenemase detected. Antimicrobial de-escalation initiated: PTZ/tazo + moxifloxacin STOPPED. Meropenem 1 g IV q8 h + linezolid 600 mg IV q12 h STARTED.	No immediate change	Targeted de-escalation based on rapid molecular diagnostics
ICU day 4	Quantitative BAL culture finalized: MRSA > 10^4^, ESBL-*Kp* > 10^5^, *Streptomyces* spp. > 10^4^. Blood cultures positive for *Candida* spp. → Candidemia confirmed. Voriconazole 200 mg IV loading, then q12 h initiated.	Fever 38.6 °C; PaO_2_/FiO_2_ 110	*Streptomyces* adjudicated as colonization (framework applied). Candidemia management with voriconazole initiated.
ICU day 6	Persistent severe hypoxemia (PaO_2_/FiO_2_ 95 despite optimization). Prone positioning initiated. Sedation optimized (propofol increased).	PaO_2_/FiO_2_ 95; HR 95 bpm; temp 37.2 °C	Prone positioning for refractory hypoxemia
ICU day 11	Gas exchange significantly improved. Prone positioning discontinued. Sedation weaned. Patient triggering ventilator appropriately.	PaO_2_/FiO_2_ 158 on FiO_2_ 0.40, PEEP 8; HR 88 bpm; temp 37.0 °C	Weaning parameters favorable; readiness for extubation assessment
ICU day 12	Extubation: successfully extubated To HFNC (FiO_2_ 0.40). Meropenem + linezolid completed (10-day course ended). Voriconazole discontinued after 7 days. Repeat blood cultures (day 12): negative.	SpO_2_ 96%–98% on HFNC FiO_2_ 0.40; HR 82 bpm; RR 18; temp 36.8 °C	Successful extubation; antimicrobials completed per protocol
Hospital day 16	Transferred from ICU to general ward. Oxygen requirement decreased to 2 L/min by nasal cannula. Pulmonary rehabilitation referral placed.	SpO_2_ 95% on 2 L/min; clinically stable	Clinical improvement; ICU discharge criteria met
Hospital day 21	Hospital discharge: discharged home on home oxygen (3 L/min continuous). Prescriptions: pulmonary rehabilitation, infectious disease follow-up appointment scheduled.	SpO_2_ 97% on 3 L/min home oxygen; clinically stable for discharge	Safe discharge home with outpatient

HFNC, high-flow nasal cannula; SIMV, synchronized intermittent mandatory ventilation; PEEP, positive end-expiratory pressure; BAL, bronchoalveolar lavage; MPCR, multiplex polymerase chain reaction. Because bronchoalveolar lavage was performed within approximately 24–36 h after endotracheal intubation, the respiratory episode is described as a lower respiratory tract infection developing during invasive mechanical ventilation, rather than definitive classical ventilator-associated pneumonia. Therapeutic modifications correspond to targeted antimicrobial optimization based on integrated microbiological and clinical assessment.

### ICU course and bronchoscopy

On ICU day 2, she developed worsening hypoxemic respiratory failure with HFNC failure (PaO_2_ 55 mmHg on FiO_2_ 1.0) and underwent orotracheal intubation followed by invasive mechanical ventilation, initially in SIMV mode and subsequently adjusted to a lung-protective ventilatory strategy according to ARDS protocol.

**FIGURE 2:**
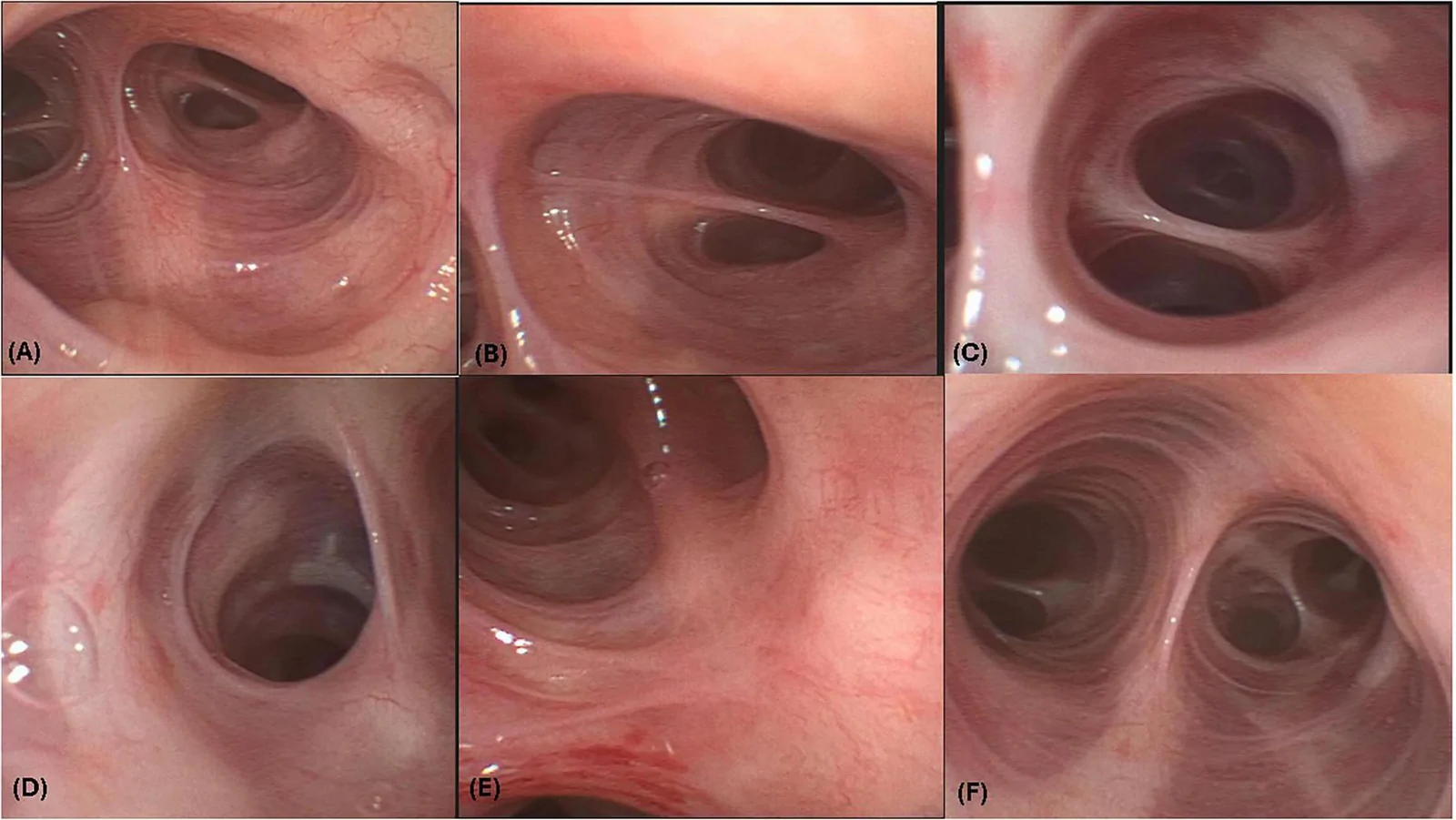
Bronchoscopic findings and bronchoalveolar lavage (ICU Day 3). Flexible bronchoscopy performed on ICU day 3 because of worsening respiratory failure, persistent fever, abundant purulent secretions, and suspicion of secondary lower respiratory tract infection developing during invasive mechanical ventilation. **(A)** Normal carina with preserved bifurcation. **(B)** Erythematous and edematous right main bronchus with abundant purulent secretions. **(C)** Partial obstruction of the right upper lobar bronchus by a mucus plug subsequently removed by suction. **(D)** Hyperemia and retained secretions within the left main bronchus. **(E)** Mild inflammatory changes in the left lower lobar bronchus. **(F)** Bronchoscopy provided a high-quality distal bronchoalveolar lavage specimen for quantitative microbiological culture and multiplex molecular analysis.

On ICU day 3, flexible bronchoscopy with bronchoalveolar lavage was performed because of suspected secondary lower respiratory tract infection, copious purulent secretions, persistent fever, worsening gas exchange, and elevated inflammatory markers. Given that BAL was obtained approximately 24–36 h after intubation, the episode is described as a lower respiratory tract infection developing during invasive mechanical ventilation rather than definitive classical ventilator-associated pneumonia. Bronchoscopy showed erythematous bronchial mucosa, abundant purulent secretions, and a mucus plug obstructing the right main bronchus, which was removed. The carina was normal with symmetric bifurcation. Additional findings included mucosal erythema in the right main bronchus, partial obstruction of the right upper lobar bronchus, hyperemia and mucus retention in the left main bronchus, and mild inflammation in the left lower lobar bronchus. BAL fluid was turbid and purulent (Figure 2).

### Microbiology and structured clinical adjudication process

Quantitative BAL cultures after 48 h demonstrated polymicrobial growth: methicillin-resistant *Staphylococcus aureus* (MRSA) > 10^4^ CFU/mL, *Klebsiella pneumoniae* > 10^5^ CFU/mL, and *Streptomyces* spp. > 10^4^ CFU/mL.

**FIGURE 3:**
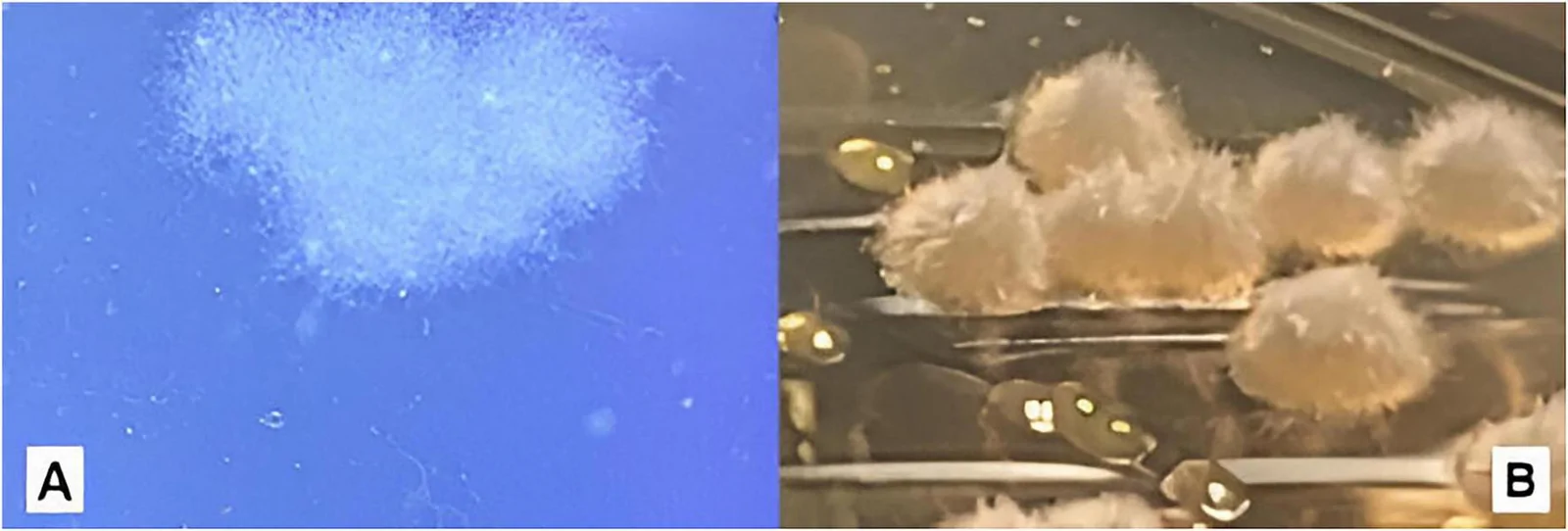
Gram stain and culture characteristics of the recovered *Streptomyces* spp. isolate. **(A)** Gram stain (oil immersion, ×1,000) demonstrating Gram-positive filamentous branching rods consistent with aerobic actinomycetes. **(B)** Aerobic culture showing dry, chalky gray-white colonies characteristic of the *Streptomyces* genus. Identification was established using routine microbiological laboratory methods; species-level molecular confirmation was not performed. Although the isolate demonstrated quantitative growth exceeding10^4^ CFU/mL, quantitative burden alone was not considered sufficient to establish pathogenicity. Interpretation was based on integration of microbiological findings with specimen quality, host characteristics, radiological assessment, therapeutic context, and overall clinical progression through the structured clinical adjudication process described in this report.

A rapid multiplex PCR pneumonia panel performed on BAL detected *S. aureus* and *K. pneumoniae*, with resistance markers consistent with MRSA (mecA) and CTX-M detected; carbapenemase markers were not detected. *Streptomyces* spp. was identified from BAL culture as an aerobic actinomycete. The BAL specimen was cultured under aerobic conditions, and Gram stain demonstrated Gram-positive filamentous branching rods consistent with aerobic actinomycetes. Genus-level identification was performed using the available microbiological identification method. Species-level confirmation by 16S rRNA sequencing was not performed. Representative Gram stain and colony morphology are shown in Figure 3. Microbiological results from quantitative BAL culture and multiplex PCR are detailed in Table 2.

**TABLE 2:** Microbiology summary: BAL quantitative culture and molecular diagnostics.

Specimen	Organism	Quantitative culture	FilmArray/MPCR result	Key resistance markers	Susceptibility summary	Adjudication
BAL	MRSA	>10^4^ CFU/mL	mecA detected	MRSA confirmed	Linezolid (S), Vancomycin (S)	Pathogen
BAL	ESBL-*Kp*	>10^5^ CFU/mL	ESBL marker detected; carbapenemase NOT detected	ESBL confirmed; no carbapenemase	Meropenem (S), Colistin (S), Fosfomycin (S)	Pathogen
BAL	*Streptomyces* spp.	>10^4^ CFU/mL	Notdetected (FilmArray panel limited)	Not tested (rare organism panel)	Susceptible to all agents tested	Colonization
Blood	*Candida* spp.	Positive (growth)	Not performed	Not applicable	Voriconazole (S)	Pathogen

BAL, bronchoalveolar lavage; MPCR, multiplex polymerase chain reaction; MRSA, methicillin-resistant *Staphylococcus aureus*; ESBL, extended-spectrum β-lactamase; CFU, colony-forming units. The FilmArray Pneumonia Panel detected *S. aureus* (mecA) and *Klebsiella pneumoniae* (CTX-M) but does not include *Streptomyces* spp. Therefore, interpretation of *Streptomyces* recovery relied on quantitative culture integrated with microbiological, clinical, radiological, therapeutic, and host-related information through the structured clinical adjudication process described in this report.

Given the recovery of *Streptomyces spp.* in a polymicrobial respiratory specimen, the isolate was interpreted through a structured clinical adjudication process rather than as an isolated microbiological result. The following domains were considered: specimen quality, quantitative burden, microbiological credibility, clinical context, host factors, therapeutic context, and clinical evolution. No individual domain was considered sufficient to establish pathogenicity or colonization.

**FIGURE 4:**
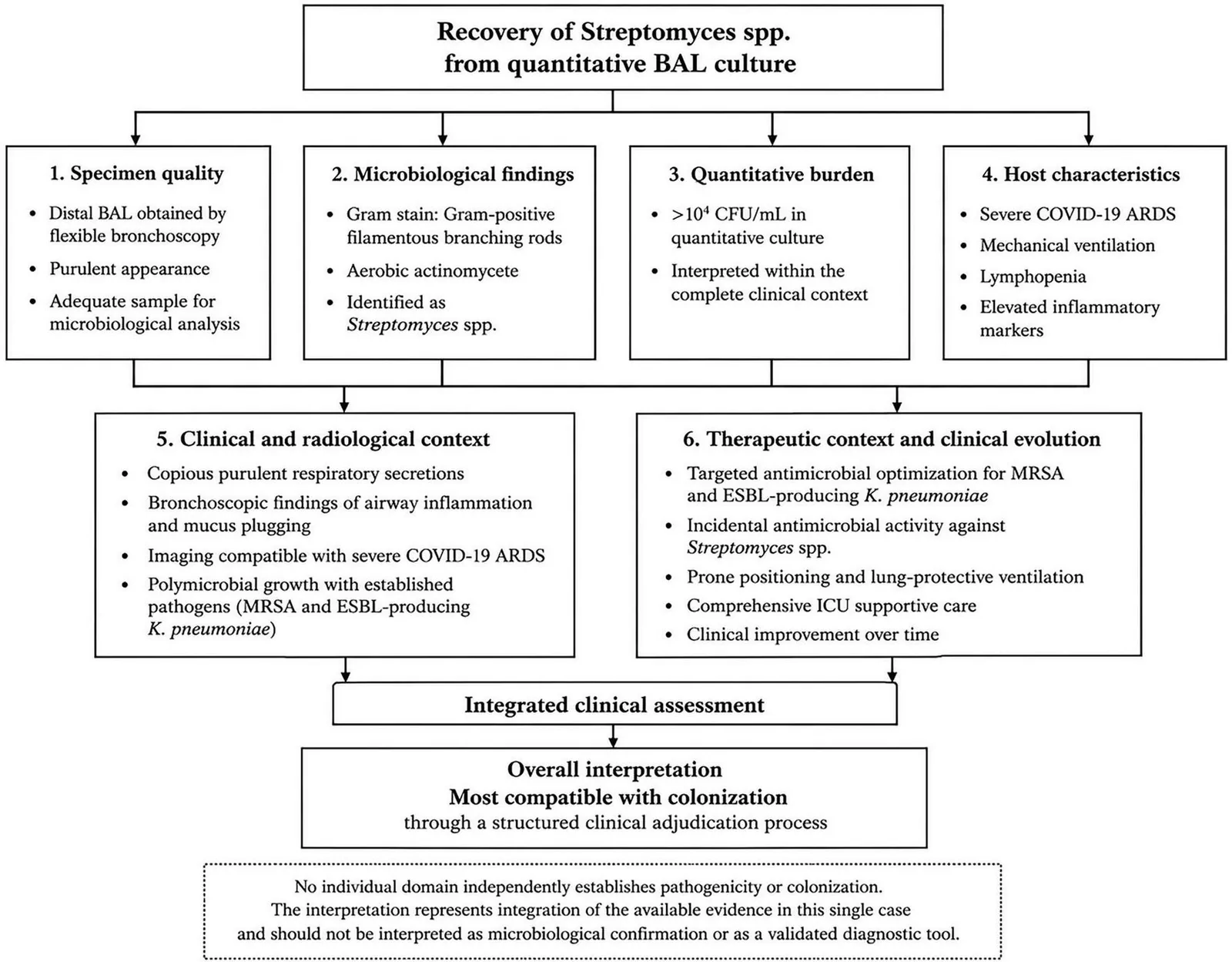
Illustrative structured clinical adjudication process applied in this case. Illustrative representation of the structured clinical adjudication process used to interpret recovery of *Streptomyces* spp. from quantitative bronchoalveolar lavage culture. Six complementary domains—including specimen quality, microbiological findings, quantitative culture, host characteristics, clinical and radiological context, and therapeutic context with clinical evolution—were integrated to support multidisciplinary clinical reasoning under conditions of microbiological uncertainty. No individual domain independently establishes pathogenicity or colonization. The overall interpretation represented an integrated clinical assessment in this single patient and was considered most compatible with colonization through a structured clinical adjudication process. This figure is intended solely as an educational and descriptive illustration of multidisciplinary clinical reasoning and should not be interpreted as microbiological confirmation, a validated diagnostic strategy, or a clinical decision-making tool.

The structured clinical adjudication process is summarized in Figure 4. Overall, recovery of *Streptomyces* spp. was considered most compatible with colonization in the context of polymicrobial lower respiratory tract infection during invasive mechanical ventilation, whereas MRSA and CTX-M–positive. *K. pneumoniae* were considered more plausible causes of the infectious syndrome based on microbiological findings, molecular confirmation, bronchoscopic findings, and clinical context. This interpretation should not be understood as microbiological confirmation of colonization.

### Antimicrobial optimization and clinical evolution

After availability of the rapid panel results on ICU day 3, empiric antibiotics were discontinued and targeted antimicrobial optimization was initiated with meropenem (1 g IV every 8 h) plus linezolid (600 mg IV every 12 h) for 10 days (ICU days 3–12). The antibiotic sequence, dosing, and rationale for antimicrobial optimization are summarized in Table 3. Susceptibility testing showed MRSA susceptible to linezolid and CTX-M– positive *K. pneumoniae* susceptible to meropenem, colistin, and fosfomycin.

**TABLE 3:** Antimicrobial therapy: chronology and dosing.

Drug	Dose	Route	Start (ICU day)	Stop (ICU day)	Total duration	Indication	Rationale for de-escalation
Piperacillin/ tazobactam	4.5 g q6 h	IV	1	3	3 days	Empiric VAP coverage (gram-negative + MRSA)	Discontinued post-FilmArray; ESBL-Kp susceptible to meropenem; MRSA susceptible to linezolid
Moxifloxacin	400 mg daily	IV/PO	1	3	3 days	Empiric atypical coverage	Discontinued post-FilmArray; not necessary given targeted coverage
Meropenem	1 g q8 h	IV	3	12	10 days	Directed therapy for ESBL-Kp	Confirmed susceptible; carbapenemase-negative; optimal coverage for ESBL
Linezolid	600 mg q12 h	IV	3	12	10 days	Directed therapy for MRSA	Confirmed susceptible; excellent lung penetration; optimal for VAP
Voriconazole	200 mg (per protocol)	IV/PO	4	10	7 days	Candidemia management (*Candida* spp., blood culture positive)	Confirmed susceptible; prolonged IV followed by PO transition

ICU day was calculated from the day of ICU admission. IV, intravenous; q6 h/q8 h/q12 h, every 6, 8, or 12 h. Targeted antimicrobial optimization refers to modification of empirical antimicrobial therapy according to rapid molecular diagnostics, quantitative bronchoalveolar lavage culture, antimicrobial susceptibility testing, and integrated clinical assessment. Caspofungin therapy was administered for 14 days after the first documented negative blood culture, consistent with current clinical practice guidelines for candidemia.

The clinical microbiology laboratory reported the isolate as susceptible to the antimicrobial agents included in the routine institutional susceptibility panel. Species-specific standardized susceptibility testing and minimum inhibitory concentration (MIC) determination were not performed. Because linezolid and meropenem provided incidental antimicrobial activity against *Streptomyces* spp., the patient should not be described as having improved without antimicrobial activity against this organism.

On ICU day 4, two consecutive peripheral blood cultures yielded *Candida* spp., confirming candidemia. Intravenous caspofungin was initiated with a 70 mg loading dose followed by 50 mg once daily, according to institutional protocol. The central venous catheter was promptly removed and replaced following the diagnosis of candidemia. Follow-up blood cultures documented microbiological clearance, and antifungal therapy continued for 14 days after the first documented negative blood culture, consistent with current clinical practice guidelines. An ophthalmologic examination was not performed because the patient remained critically ill under invasive mechanical ventilation throughout the acute phase of illness.

The patient required ongoing invasive mechanical ventilation with intensive airway clearance. On ICU day 6, prone positioning was initiated for persistent severe hypoxemia (PaO_2_/FiO_2_ 95). By ICU day 11, gas exchange improved (PaO_2_/FiO_2_ 158 on FiO_2_ 0.40, PEEP 8). She was extubated on ICU day 12 and transitioned to HFNC (FiO_2_ 0.40). She was transferred to the general ward on hospital day 16 and discharged on hospital day 21 with SpO_2_ 97% on home oxygen (3 L/min via nasal cannula), outpatient pulmonary rehabilitation, and infectious disease follow-up.

Because clinical improvement occurred alongside multiple concurrent interventions— including targeted antimicrobial optimization, incidental antimicrobial activity against *Streptomyces* spp., candidemia management, lung-protective ventilation, prone positioning, and comprehensive ICU supportive care—the favorable clinical course should not be interpreted as evidence supporting either pathogenicity or colonization of *Streptomyces* spp.

### Post-discharge functional assessment

Pulmonary function testing at 8 weeks post-discharge showed mild restrictive ventilatory impairment with reduced diffusing capacity (FVC 75% predicted, DLCO 70% predicted). Results are provided in Supplementary Table 1.

Cardiopulmonary exercise testing performed 6 months after discharge showed severe exercise limitation (peak VO_2_ 635 mL/min, 41% predicted) and ventilatory inefficiency (VE/VCO_2_ 47.8 predicted), consistent with post-ARDS functional impairment. CPET parameters are detailed in Supplementary Table 2. The patient was referred for intensive cardiopulmonary rehabilitation.

## Discussion

This case illustrates the clinical complexity of interpreting Streptomyces spp. recovered from bronchoalveolar lavage in a polymicrobial lower respiratory tract infection developing during invasive mechanical ventilation. Rather than proposing a diagnostic algorithm, this report describes a structured clinical adjudication process in which microbiological findings were integrated with host characteristics, radiological assessment, therapeutic context, and clinical progression to support antimicrobial decision-making under conditions of microbiological uncertainty. To our knowledge, few published reports have described such an integrated clinical reasoning process in patients with recovery of *Streptomyces* spp. from lower respiratory tract specimens. (4, 5)

Lower respiratory tract infections developing during invasive mechanical ventilation frequently present with polymicrobial microbiological findings in which well-recognized pathogens coexist with microorganisms whose clinical significance is uncertain. In these situations, interpretation should rely on integration of microbiological, clinical, radiological, and host-related information rather than on culture results alone (8, 9). Most published cases have occurred in markedly immunocompromised hosts, whereas recovery from respiratory specimens more commonly represents contamination or colonization. Nevertheless, critically ill patients with severe viral ARDS (10, 11) may develop profound immune dysregulation, airway epithelial injury, and polymicrobial respiratory infections, making interpretation of uncommon environmental microorganisms substantially more challenging (1, 2, 11). *Streptomyces* spp. are ubiquitous environmental aerobic actinomycetes that only rarely cause invasive disease, and recovery from respiratory specimens more commonly represents contamination or colonization than true infection (4–6).

Accordingly, the principal clinical question is not whether *Streptomyces* spp. can cause pulmonary infection, but whether the organism is likely to have contributed meaningfully to the infectious process in an individual patient when interpreted within the complete clinical context. This distinction is particularly relevant for antimicrobial stewardship because unnecessary expansion of antimicrobial therapy may increase toxicity, ecological impact, and selection of antimicrobial resistance without improving clinical outcomes. (12, 13)

To facilitate interpretation of *Streptomyces spp.* recovered from distal bronchoalveolar lavage, we applied a structured clinical adjudication process integrating multiple complementary domains, including specimen quality, microbiological findings, quantitative culture, clinical and radiological context, host characteristics, therapeutic context, and overall clinical evolution. Rather than functioning as a diagnostic algorithm, this process represents an educational illustration of multidisciplinary clinical reasoning in a situation of microbiological uncertainty (5, 12).

Importantly, none of these domains independently establishes either pathogenicity or colonization. Instead, each contributes complementary information that should be interpreted collectively within the overall clinical context (14).

In the present case, interpretation was influenced not only by microbiological findings but also by the presence of alternative respiratory pathogens with established pathogenic potential, bronchoscopic findings, radiological assessment, host characteristics, and the therapeutic context.

Clinical evolution was considered supportive contextual information rather than diagnostic evidence because recovery may have resulted from multiple concurrent interventions, including targeted antimicrobial optimization directed against MRSA and ESBL-producing *K. pneumoniae*, incidental antimicrobial activity against *Streptomyces* spp., lung-protective ventilation, prone positioning, candidemia management, and comprehensive intensive care support. Consequently, favorable clinical evolution should not be interpreted as microbiological evidence supporting either pathogenicity or colonization.

Conventional quantitative culture of bronchoalveolar lavage generally requires 48–72 h before complete microbiological characterization becomes available (7). In contrast, multiplex PCR rapidly identified MRSA and CTX-M–producing *K. pneumoniae* together with their principal resistance determinants within hours. When interpreted alongside quantitative culture and the overall clinical assessment, these findings facilitated timely targeted antimicrobial optimization directed toward pathogens considered clinically relevant.

Nevertheless, rapid molecular techniques should be interpreted as complementary diagnostic tools and not as substitutes for comprehensive clinical assessment, particularly when uncommon microorganisms not included in commercial molecular panels are recovered by conventional culture (7).

Polymicrobial bronchoalveolar lavage findings frequently complicate antimicrobial decision-making, particularly when uncommon environmental organisms are recovered together with established respiratory pathogens. In this case, MRSA and CTX-M–positive (15, 16) *K. pneumoniae* represented more plausible causes of the infectious syndrome based on their recognized pathogenic potential, molecular confirmation, quantitative burden, and concordance with bronchoscopic findings. *Streptomyces* spp., despite quantitative growth > 10^4^ CFU/mL, was interpreted cautiously because quantitative burden alone does not establish pathogenicity (14, 17, 18).

The overall findings were considered most compatible with colonization through a structured clinical adjudication process, without microbiological confirmation.

The concurrent episode of candidemia further illustrates the complexity of infectious complications in critically ill patients with severe COVID-19 ARDS. Two consecutive blood cultures yielded *Candida* spp., and the patient received caspofungin with central venous catheter removal and replacement, followed by 14 days of therapy after the first documented negative blood culture, consistent with current clinical practice guidelines. This episode reinforces the need for continuous reassessment as new microbiological data emerge during ICU care (19).

Despite survival and respiratory stabilization, post-discharge assessment documented persistent functional impairment, including reduced diffusing capacity at 8 weeks and peakVO_2_41% predicted at 6 months. These findings are consistent with the long-term physical limitations described in ARDS survivors and support the importance of structured rehabilitation and follow-up after critical illness (20, 21).

This report has several important limitations inherent to its descriptive design. As a single case report, its observations should not be generalized, and no causal relationship can be established between the structured clinical adjudication process, targeted antimicrobial optimization, and the patient’s clinical outcome.

The favorable clinical evolution was likely influenced by multiple concurrent interventions, including lung-protective mechanical ventilation, prone positioning, antimicrobial therapy directed against MRSA and ESBL-producing *K. pneumoniae*, incidental antimicrobial activity against *Streptomyces* spp., candidemia management, and comprehensive intensive care support, in addition to the natural evolution of COVID-19 ARDS. Consequently, clinical improvement should not be interpreted as evidence supporting either pathogenicity or colonization of *Streptomyces* spp.

Additional immunological investigations, including CD4 + T-cell count, immunoglobulin levels, complement assays, and cytokine profiling, were not performed as part of routine clinical management. Consequently, a more comprehensive characterization of the patient’s immune status was not possible.

Furthermore, interpretation of lower respiratory tract infection developing during invasive mechanical ventilation in patients with severe viral ARDS remains challenging, particularly when polymicrobial bronchoalveolar lavage cultures are obtained early during the course of invasive ventilation.

Microbiological certainty was also limited because *Streptomyces* spp. was identified only at the genus level using routine laboratory methods. Species-level molecular confirmation was not available, and serial bronchoalveolar lavage cultures were not performed to assess microbiological persistence.

Quantitative culture thresholds should likewise be interpreted cautiously, as bacterial burden alone cannot distinguish colonization from infection and may be influenced by sampling technique, timing of specimen collection, and previous antimicrobial exposure. Moreover, currently available multiplex PCR respiratory panels do not detect *Streptomyces* spp., requiring integration of conventional microbiology with clinical assessment (7). In addition, antimicrobial susceptibility testing for the *Streptomyces* isolates reflected routine clinical laboratory practice. Species-specific standardized susceptibility testing and MIC determination were not available; therefore, the reported susceptibility profile should be interpreted within this limitation.

Finally, the concurrent episode of candidemia and the complexity of critical illness further limit attribution of the observed clinical course to any individual microorganism or therapeutic intervention. Accordingly, the structured clinical adjudication process presented in this report should be interpreted as an educational and descriptive illustration of multidisciplinary clinical reasoning applied to this particular case, rather than as a validated diagnostic strategy or clinical decision-making tool.

The present case highlights the importance of integrating microbiological findings with the broader clinical context when uncommon microorganisms are recovered from lower respiratory tract specimens in critically ill patients. Rather than relying on quantitative culture or organism identification alone, interpretation should incorporate specimen quality, host characteristics, radiological assessment, therapeutic context, and overall clinical progression. Although this report does not establish a diagnostic strategy, it illustrates how a structured clinical adjudication process may facilitate multidisciplinary clinical reasoning and support targeted antimicrobial optimization under conditions of microbiological uncertainty (12, 13).

This case illustrates the application of a structured clinical adjudication process to interpret the recovery of *Streptomyces* spp. from bronchoalveolar lavage in a patient with severe COVID-19 ARDS complicated by polymicrobial lower respiratory tract infection during invasive mechanical ventilation.

Integration of quantitative bronchoalveolar lavage culture with rapid multiplex PCR contributed to timely microbiological assessment and supported targeted antimicrobial optimization directed toward pathogens considered clinically relevant. Interpretation of *Streptomyces* spp. required integration of microbiological findings with host characteristics, radiological assessment, therapeutic context, and overall clinical progression, rather than reliance on any single microbiological parameter.

The structured clinical adjudication process described in this report illustrates how microbiological findings, host factors, radiological assessment, therapeutic context, and clinical evolution can be integrated when uncommon respiratory microorganisms are recovered in critically ill patients. Rather than establishing pathogenicity or providing microbiological confirmation of colonization, this approach emphasizes multidisciplinary clinical interpretation under conditions of diagnostic uncertainty. Whether similar structured approaches improve consistency in clinical decision-making should be evaluated in future studies involving larger numbers of patients.

## Conclusion

This case describes the application of a structured clinical adjudication process integrating microbiological findings, host characteristics, radiological assessment, therapeutic context, and clinical evolution during management of a polymicrobial lower respiratory tract infection developing during invasive mechanical ventilation. The report should be regarded as a descriptive clinical illustration rather than validation of a diagnostic strategy. Additional prospective studies are needed to determine the reproducibility and clinical utility of similar structured approaches in patients with uncommon respiratory microorganisms.

## Data Availability

The raw data supporting the conclusions of this article will be made available by the authors, without undue reservation.

## Supplementary material

Supplementary Table 1 Pulmonary function testing 8 weeks after discharge. Pred, predicted; %Pred, percent predicted; Pre/Post, pre-/post- bronchodilator (as reported). FVC, forced vital capacity; FEV1, forced expiratory volume in 1 second; FEF25–75, forced expiratory flow 25%–75%; PEF, peak expiratory flow; TLC, total lung capacity; VC, vital capacity; RV, residual volume; FRC, functional residual capacity; ERV, expiratory reserve volume; IC, inspiratory capacity; DLCO, diffusing capacity for carbon monoxide; VA, alveolar volume; KCO, transfer coefficient. Values are presented as reported by the pulmonary laboratory

Supplementary Table 2 (CPET, 6 months) cardiopulmonary exercise testing at 6 months after discharge. Pred, predicted; AT, anaerobic threshold; RCP, respiratory compensation point; HR, heart rate; VO_2_, oxygen uptake; VCO_2_, carbon dioxide output; RER, respiratory exchange ratio; VE, minute ventilation; BF, breathing frequency; BPsys/BPdia, systolic/diastolic blood pressure; BR, breathing reserve; VE/VO_2_ and VE/VCO2, ventilatory equivalents for oxygen and carbon dioxide; HRR, heart rate reserve. Percentages are calculated relative to predicted or maximal values as indicated.
